# Supplementary material for: Perceived Control and Work-Related Stress Mediate the Effects of Grit on Depression among Employees
Source: Brain Sci. 2022 Dec 20;13(1):9. doi: 10.3390/brainsci13010009 (PMC9856448; doi:10.3390/brainsci13010009)
Supplement: Supplementary file 1 [file brainsci-13-00009-s001.zip › brainsci-2058304-supplementary.pdf]

Table S1. Inter-correlation of subscales of KOSS

	Grit 1	Grit 2	1.	2.	3.	4.	5.	6.	7.	8.	CES-D
Grit 1 (Passion)	-										
Grit 2 (Perseverance)	0.495**	-									
1. Difficult physical environment	-0.156**	-0.217**	-								
2. High job demand	-0.264**	-0.145**	0.242**	-							
3. Insufficient job control	-0.172**	-0.327**	0.218**	0.079**	-						
4. Inadequate social support	-0.199**	-0.266**	0.248**	0.240**	0.289**	-					
5. Job insecurity	-0.218**	-0.229**	0.287**	0.234**	0.1033**	0.324**	-			-	
6. Organizational injustice	-0.305**	-0.351**	0.397**	0.379**	0.387**	0.517**	0.372**	-			
7. Lack of reward	-0.327**	-0.437**	0.328**	0.330**	0.466**	0.536**	0.352**	0.723**	-		
8. Discomfort in occupational climate	-0.319**	-0.320**	0.363**	0.352**	0.293**	0.458**	0.404**	0.592**	0.571**	-	
CES-D	-0.440**	-0.438**	0.270**	0.399**	0.276**	0.393**	0.369**	0.495**	0.549**	0.478**	-

KOSS, Korean Occupational Stress Scale; CES-D, Center for Epidemiologic Studies Depression Scale.
** $p<0.001$.

Table S2. Standardized indirect effects and 95% confidence intervals of each pathway between grit and depression mediated by occupational stress.

Pathway	Estimate	95% Confidence Interval	
		Lower	Upper
Passion → Occupational stress → Depression			
Passion → Difficult physical environment → Depression	-0.001	-0.003	0.002
Passion → High job demand → Depression	-0.036	-0.040	-0.031
Passion → Insufficient job control → Depression	-0.003	-0.005	0.000
Passion → Inadequate social support → Depression	-0.015	-0.019	-0.011
Passion → Job insecurity → Depression	-0.026	-0.031	-0.022
Passion → Organizational injustice → Depression	-0.010	-0.016	-0.003
Passion → Lack of reward → Depression	-0.070	-0.079	-0.062
Passion → Discomfort in occupational climate → Depression	-0.026	-0.032	-0.020
Perseverance → Occupational stress → Depression			
Perseverance → Difficult physical environment → Depression	0.001	-0.002	0.005
Perseverance → High job demand → Depression	-0.025	-0.030	-0.021
Perseverance → Insufficient job control → Depression	0.001	-0.004	0.006
Perseverance → Inadequate social support → Depression	-0.019	-0.024	-0.013
Perseverance → Job insecurity → Depression	-0.028	-0.034	-0.024
Perseverance → Organizational injustice → Depression	-0.014	-0.022	-0.007
Perseverance → Lack of reward → Depression	-0.087	-0.098	-0.077
Perseverance → Discomfort in occupational climate → Depression	-0.031	-0.037	-0.025

Confidence intervals, 95%; number of bootstrap samples, 5000.

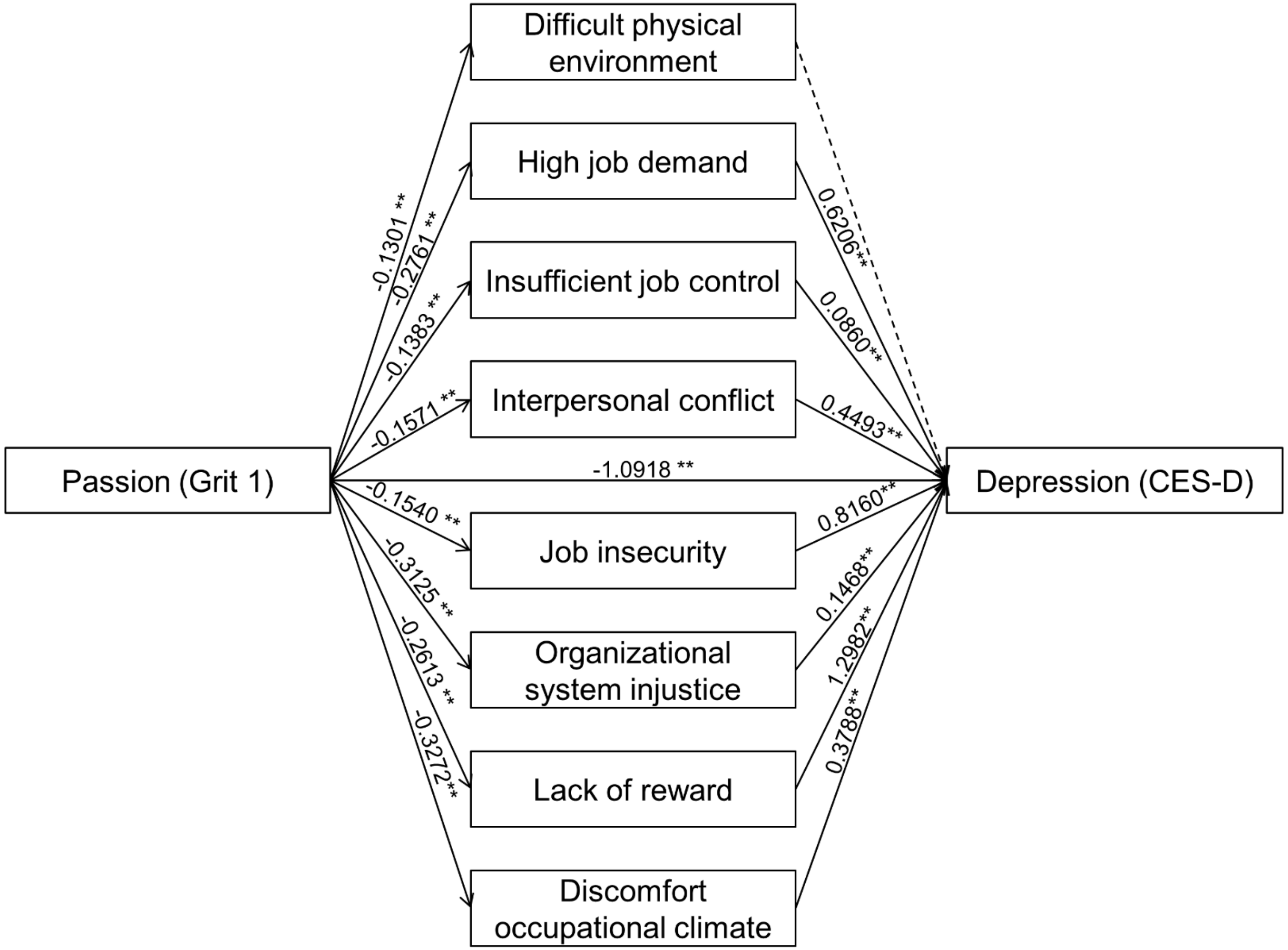

Figure S1. Mediation model of subtypes of occupational stress between passion and depression. All path coefficients are standardized. **P< 0.001

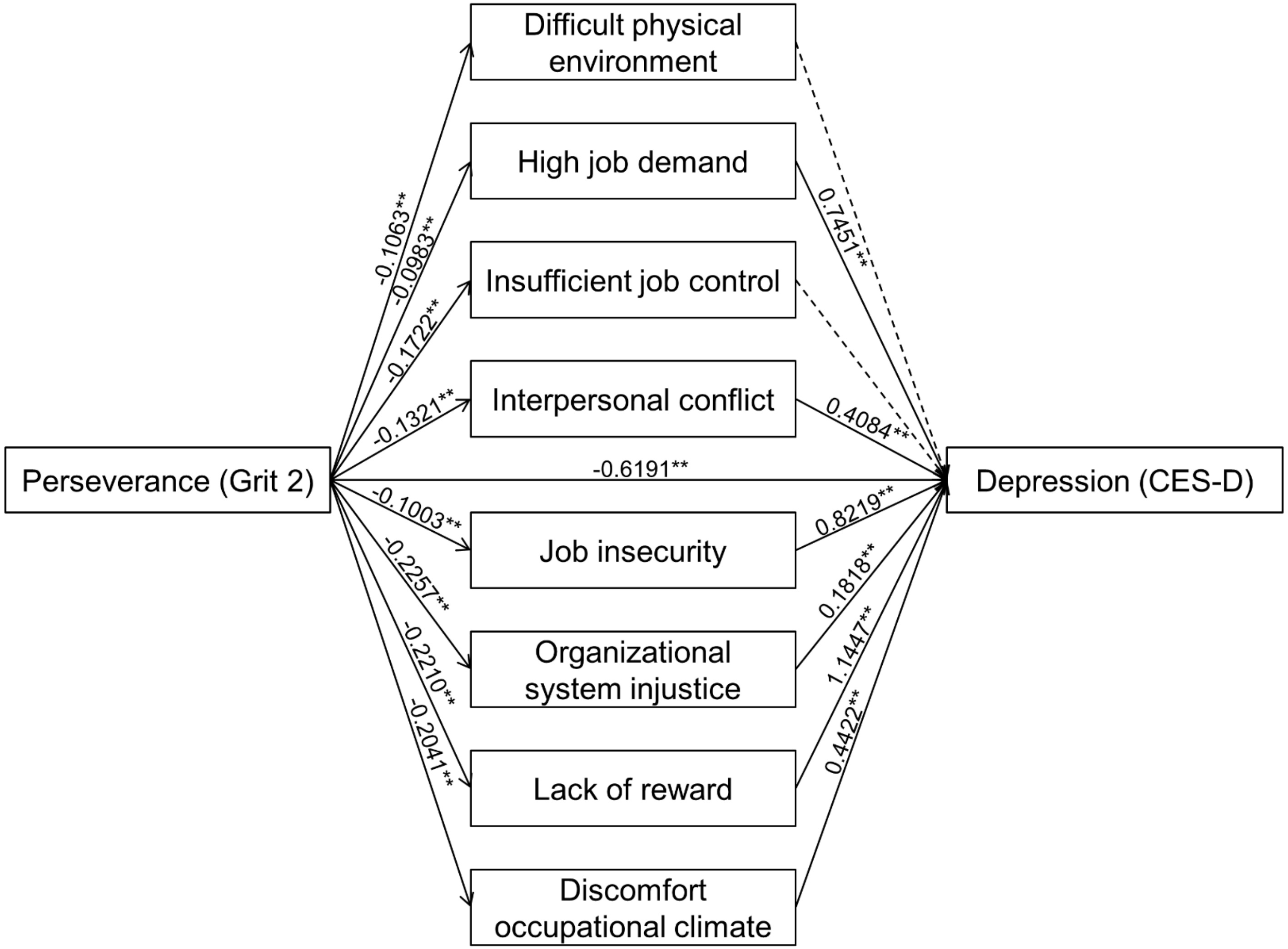

Figure S2. Mediation model of subtypes of occupational stress between perseverance and depression. All path coefficients are standardized. **P< 0.001
